# Cosinor-based rhythmometry

**DOI:** 10.1186/1742-4682-11-16

**Published:** 2014-04-11

**Authors:** Germaine Cornelissen

**Affiliations:** 1 Halberg Chronobiology Center, University of Minnesota, 420 Delaware Street SE, 55455 Minneapolis, MN, USA

**Keywords:** Chronobiology, Chronome, Circadian, Cosinor, External information, Regression, Rhythm parameters, Stationarity

## Abstract

A brief overview is provided of cosinor-based techniques for the analysis of time series in chronobiology. Conceived as a regression problem, the method is applicable to non-equidistant data, a major advantage. Another dividend is the feasibility of deriving confidence intervals for parameters of rhythmic components of known periods, readily drawn from the least squares procedure, stressing the importance of prior (external) information. Originally developed for the analysis of short and sparse data series, the extended cosinor has been further developed for the analysis of long time series, focusing both on rhythm detection and parameter estimation. Attention is given to the assumptions underlying the use of the cosinor and ways to determine whether they are satisfied. In particular, ways of dealing with non-stationary data are presented. Examples illustrate the use of the different cosinor-based methods, extending their application from the study of circadian rhythms to the mapping of broad time structures (chronomes).

## Introduction

Non-random variations are found as a function of time at the cellular level, in tissue culture, as well as in multi-cellular organisms at different levels of physiologic organization [1]. Multi-frequency rhythms usually account for a sizeable portion of the variability [2]. While there is presently much interest in studying circadian rhythms, the biological time structure covers many different ranges of periods beyond the 24-hour day, from fractions of seconds in single neurons to seconds in the cardiac and respiratory cycles, and a few hours in certain endocrine functions. Cycles with periods of about a week, about a month, and about a year are also ubiquitous, as are some other newly discovered cycles with periods of about 5 and 16 months, and much longer periods [3].

The partly built-in nature of circadian rhythms [4,5] is now widely accepted, as is the fact that they are amenable to synchronization by cycles in the environment (e.g., lighting and feeding schedules) [6]. More generally, environmental geophysical cycles such as the day-light cycle, the tides, the phases of the moon, the seasons, as well as a host of other cycles shared between living organisms and the environment in which they evolved, all serve as synchronizers for partly endogenous rhythms [7,8].

The application of chronobiology and its concepts to biology and medicine depends upon the quantitative evaluation of data collected as a function of time. The inclusion of time as a primordial factor in chronobiological investigations broadens the scope of methods for data analysis. The methods presented herein serve the purposes of rhythm detection and parameter estimation, with applications in the early diagnosis of altered rhythm characteristics indicative of a heightened risk, the optimization of treatment by timing, and a wider understanding of how our physiology is influenced by our environment.

### Data collection and study design

Before turning to the methodology itself, it is important to consider aspects of data collection and study design [9] bearing on the choice of analytical tools used for data analysis. Biological data (Y_i_, i = 1, 2, …, N) are typically obtained by having a clock trigger the system (instrument, sensor) to measure a biological variable, yielding a set of data at discrete sampling times (t_i_, i = 1, 2, …, N). Whether the variable examined is discrete (e.g., mitotic counts) or continuous (e.g., oral temperature), the numerical values attributed to Y_i_ are limited in accuracy and precision by the instrumentation used. Any finite variation of a biological system takes place during a non-zero time interval rather than instantaneously. In terms of data analysis, this means that there is a cut-off frequency f_S_ beyond which the spectrum of the biological variations is practically zero [10]. Whether the transducer used to measure a given biological variable is analog or digital, it takes a certain time for it to respond and deliver a reading, so that rhythms with periods shorter than this response time cannot be assessed, and rhythms with a period close to it will be distorted [10]. In other words, a cut-off frequency f_T_ can be defined as the minimal frequency such that for f > f_T_ the output signal remains practically constant (no variation in the data can be assessed). This means that too dense measurements are redundant and do not bring additional information. In the case of equidistant data obtained at Δt intervals, it has been recommended to choose Δt ≤ 1/4f_T_ to assure a good approximation of Y(t) [11].

For chronobiological applications, this sampling requirement (to be able to reconstruct changes as a function of time in the context of the theory of signal processing) is often difficult to meet. Instead, sampling is used in its statistical meaning, where it refers to the selection of a few items from a population to draw inferences for that population. In terms of a data series, the selection of a sampling interval Δt > 1/f_T_ allows only some features of the biological variations to be assessed. In the absence of external information, for data collected over an observation span T, only oscillations with periods in the range of T up to 2Δt can be assessed. The resolution with which a signal’s period can be determined also depends on T: in frequency terms, the smallest difference in frequency between two distinct signals is 1/T. The highest (no longer assessable) frequency, 1/2Δt, is called the Nyquist frequency, f_N_. Within the field of information theory, this is known as the Nyquist-Shannon theorem, which states that if a function Y(t) contains no frequencies higher than f_N_, it is completely determined by sampling Y(t) at intervals of 1/2f_N_.

Ostle [12] defines the design of an experiment as the complete sequence of steps taken ahead of time to ensure that the appropriate data are obtained in a way which allows for an objective analysis leading to valid inferences with respect to the stated problem. These steps include the statement of objectives, the formulation of hypotheses, and the choice of design and experimental procedure and of the statistical methods to be used. Principles underlying experimental designs rely on replication, randomization and control. Replication relates to repeated measurements to obtain an estimate of uncertainty (experimental error or noise) used to derive statistical significance (P-values) and confidence intervals. Noise originates from variations in the biological system considered not to be part of the deterministic portion of the signal, from errors external to the system (errors of experimentation, of observation, and/or of measurement), and from the transducer and sampler (instrumentation error). Reducing the experimental error increases the precision of experiments. Randomization is an important aspect of study design that allows researchers to proceed as though the assumption of independence of the observation errors is true, which is critical in applying a test of significance. Although randomization cannot guarantee independence, it reduces the correlation that tend to characterize errors associated with experimental material (experimental unit or data) adjacent in space or time, while also improving accuracy. Control relates to the amount of balancing, blocking and grouping of the experimental units [12].

The number of replications needed for a given probability of detecting a given difference with statistical significance depends on the standard error per experimental unit [13]. This means that small sample sizes can easily detect large differences, whereas small differences require larger sample sizes. When dealing with rhythmic variables, a sizeable portion of the variance stems from the rhythmic variation. Assessing the rhythmic behavior is thus important to reduce the error term. One important feature of chronobiological study designs is that rhythm stage is often the primary factor, as when assessing the relative efficacy or toxicity of a given treatment administered at different stages of the circadian rhythm. The power of testing for a time effect is usually only slightly affected by the number of timepoints considered when results are analyzed by cosinor, but not when performing an analysis of variance. This difference in approach accounts in part for the controversy between classical designs advocating fewer test groups [14] and chronobiological designs recommending at least 6 timepoints per cycle [15-17].

In the framework of chronobiological study designs, three kinds of data can be distinguished, which determine the choice of method for their analysis and how the results can be interpreted. Longitudinal sampling corresponds to obtaining data on the same individual (experimental unit) as a function of time. One example is the around-the-clock monitoring of blood pressure at about 30-minute intervals for 7 days. Results apply to this particular individual. Transverse (cross-sectional) sampling consists of obtaining only one value per individual (experimental unit), different individuals providing data at the same or different sampling times. Time series of survival times are one example of transverse data. When individuals represent a random sample of a given population, results can be generalized for that population. Hybrid (linked cross-sectional) sampling consists of taking a few serial measurements from several individuals (experimental units). For instance, circulating prolactin is determined at 20-minute intervals for 24 hours in women at low or high familial risk of developing breast cancer later in life. The circadian rhythm can be determined for each woman and summarized across all women in each group for assessing any difference as a function of breast cancer risk [18]. When individuals represent a random sample of their respective populations, results can be generalized to these populations.

Quite generally, but particularly when sampling is performed on more than a single individual, it is important that they are synchronized. Synchronizers (environmental periodicities determining the temporal placement of biological rhythms) serve this purpose. The rest-activity schedule or the light–dark regimen are effective synchronizers and can be used to determine a reference time (such as time of awakening or light onset in preference to clock hours such as local midnight). Staggered lighting regimens have been used to facilitate data collection in the experimental laboratory [19], making it possible to collect data over several days [20]. Marker rhythms [21] are a useful check of whether synchronization has been achieved, further providing an internal reference. Activity, temperature, heart rate and blood pressure are some useful marker variables that can easily be monitored longitudinally. For instance, blood pressure has been used to guide the timing of administration of anti-hypertensive medication while also providing information regarding the patient’s response to treatment [22].

### Summary statistics

Before proceeding with any data analysis, it is recommended to first plot the data as a function of time. Such a chronogram can be informative in several ways. The presence of obvious rhythmicity may be recognized and its relative prominence as compared to the noise may be qualitatively (macroscopically) assessed. When sampling covers several cycles, some measure of the cycle-to-cycle variability can be gained. The presence of any increasing or decreasing trends can be observed, as is the existence of any outliers. After curve fitting, a chronogram of residuals can also provide valuable information regarding the adequacy of the model, and the need for data transformation.

A histogram should also be prepared to obtain an estimate of the mean value with its standard deviation, and to check on the assumption of normality. For instance, a long-tailed distribution is indicative of the need for data transformation. Alternatively, the use of robust methods (such as those based on ranks; [23]) may be indicated.

When prior information suggests the presence of a rhythm with known period, stacking the data over a single cycle reduces the noise and reveals the rhythm’s waveform. Historically, this approach was used by Franz Halberg to resolve confusing variability in blood eosinophil counts [24-26]. It was also instrumental in showing that the circadian rhythm in core temperature of Fischer rats persisted after the bilateral lesioning of the suprachiasmatic nuclei, albeit with a reduced amplitude and a phase advance [27,28]. It should be noted, however, that stacking the data over an assumed period may yield spurious results if the signal’s period differs from its assumed value. For this reason, it is highly recommended to analyze the original data first before proceeding with any stacking. For instance, it is not uncommon to present data by calendar month, even when the data have been collected over several years. This procedure limits the ability to resolve any periodic signal with a period different from precisely 1 year or its harmonic terms (6, 4, 3 months, …). Stacking the data over a period that has been validated can be complemented by an analysis of variance testing for a time effect when data are binned into a given number of classes of equal duration covering the full cycle (e.g., six 4-hourly classes covering 24 hours). An F-test then serves for testing the equality of class means. While this procedure remains applicable for non-equidistant data, the result depends on the choice of the number of classes used for binning and on the choice of the reference time [29].

### Single cosinor

Historically, the single cosinor was developed to analyze short and sparse data series [2,30-32]. Periodograms and classical spectra originally used in chronobiology [33,34] required the data to be equidistant and to cover more than a single cycle. Whereas some spectral analysis techniques are now available to analyze non-equidistant data [35-37], algorithms available in most software packages remain limited to the case of equidistant data.

Least squares procedures do not have this limitation. They are thus useful in curve-fitting problems, where it is desirable to obtain a functional form that best fits a given set of measurements. Although periodic regression presents its own limitations, being sensitive to outliers and not having any constraint to conserve the variance in the data, it possesses two important features: first, when data are equidistant, results at Fourier frequencies are identical to those of the discrete Fourier transform [38]; and second, it advantageously uses prior information. Thus, after the existence of ubiquitous circadian rhythms was demonstrated, it was possible to apply the single cosinor method in many experiments aimed at determining the times of highest efficacy and lowest toxicity in response to a variety of drugs and other stimuli by fitting a 24-hour cosine curve to 6 values, 4 hours apart, each value representing the number of experimental animals that survived a given intervention applied at one of the 6 timepoints when overall about 50% of the animals had died. These results led to the fields of chronopharmacy and chronotherapy [39-42].

### Single-component cosinor

**Figure 1:**
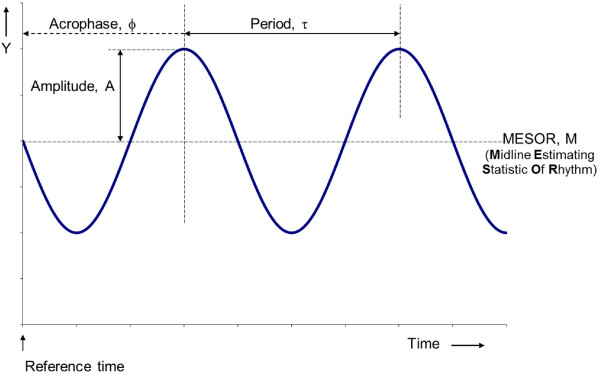
**Definition of rhythm characteristics.** The MESOR is a rhythm-adjusted mean; the double amplitude (2A) is a measure of the extent of predictable change within a cycle; the acrophase is a measure of the timing of overall high values recurring in each cycle, expressed in (negative) degrees in relation to a reference time set to 0°, with 360° equated to the period; and the period is the duration of one cycle. © Halberg Chronobiology Center.

Notably in studies of circadian rhythms, it is indeed possible to assume that the period is known, being synchronized to the external 24-hour cycle. The regression model for a single component can be written as

(1) $$Y\left(t\right)=M+\text{Acos}\left(2\mathrm{\pi t}/\tau +\phi \right)+e\left(t\right)$$

where M is the MESOR (Midline Statistic Of Rhythm, a rhythm-adjusted mean), A is the amplitude (a measure of half the extent of predictable variation within a cycle), ϕ is the acrophase (a measure of the time of overall high values recurring in each cycle), τ is the period (duration of one cycle), and e(t) is the error term (Figure 1).

When τ can be assumed known, using well-known trigonometric angle sum identity, the model can be rewritten as

(2) $$Y\left(t\right)=M+\mathrm{\beta x}+\mathrm{\gamma z}+e\left(t\right)$$

where

$$\beta =\text{Acos}\phi ;\gamma =‒\text{Asin}\phi ;x=\text{cos}\left(2\mathrm{\pi t}/\tau \right);z=\text{sin}\left(2\mathrm{\pi t}/\tau \right).$$

The principle underlying the least squares method is the minimization of the residual sum of squares (RSS), that is the sum of squared differences between measurements Y_i_ (obtained at times t_i_, i = 1, 2, …, N) and the values estimated from the model at corresponding times

(3) $$\text{RSS}=\sum_{i}{[Y}_{i}-\left(\hat{M}+\hat{\beta }x_{i}+\hat{\gamma }z_{i}\right)]{}^{2}$$

This approach is valid when all individual standard deviations are equal, as is often the case.

Estimates for M, β, and γ are obtained by solving the normal equations, obtained by expressing that RSS is minimal when its first-order derivatives with respect to each parameter are zero.

The normal equations are

(4) $$\begin{array}{l} \sum Y_{i}=\mathrm{MN}+\beta \sum x_{i}+\;\gamma \sum z_{i}\\ \sum Y_{i}x_{i}=M\sum x_{i}+\beta \sum {x_{i}}^{2}+\gamma \sum x_{i}z_{i}\\ \sum Y_{i}z_{i}=M\sum z_{i}+\beta \sum x_{i}z_{i}+\gamma \sum {z_{i}}^{2} \end{array}$$

or in matrix form

(5) $$\left(\begin{array}{c} \sum Y_{i}\\ \sum Y_{i}x_{i}\\ \sum Y_{i}z_{i} \end{array}\right)=\left(\begin{array}{ccc} N & \sum x_{i}\; & \sum z_{i}\\ \sum x_{i}\; & \sum {x_{i}}^{2}\; & \sum x_{i}z_{i}\;\\ \sum z_{i} & \sum x_{i}z_{i} & {\sum z_{i}}^{2} \end{array}\right)\;\left(\begin{array}{c} M\\ \beta \\ \gamma \end{array}\right)\;\mathrm{or}\;d=\mathrm{Su}$$

Estimates of M, β and γ (or vectorially, u) are thus obtained as

(6) $$\hat{u}=S^{‒1}d$$

Estimates for the amplitude and acrophase can be derived from the estimates of β and γ by the following relations

(7) $$\begin{array}{l} \hat{A}={\left({\hat{\beta }}^{2}+{\hat{\gamma }}^{2}\right)}^{1/2}\\ \hat{\phi }=\arctan\left(‒\hat{\gamma }/\hat{\beta }\right)+K\pi \;\mathrm{where}\;K\;\mathrm{is}\;\mathrm{an}\;\mathrm{integer} \end{array}$$

The correct value of $\hat{\phi }$ is determined by taking into account the signs of $\hat{\beta }\;\mathrm{and}\;\hat{\gamma }$.

For rhythm detection, the total sum of squares (TSS) is partitioned into the sum of squares due to the regression model (MSS) and the residual sum of squares (RSS). TSS is the sum of squared differences between the data and the arithmetic mean. MSS is the sum of squared differences between the estimated values based on the fitted model and the arithmetic mean. As noted above, RSS is the sum of squared differences between the data and the estimated values from the fitted model.

(8) $$\mathrm{TSS}=\mathrm{MSS}+\mathrm{RSS}\;\mathrm{or}\;\sum {\left(Y_{i}‒\bar{Y}\right)}^{2}=\sum {\left({\hat{Y}}_{i}‒\bar{Y}\right)}^{2}+\sum {\left(Y_{i}‒{\hat{Y}}_{i}\right)}^{2}$$

The model is statistically significant when the model sum of squares is large relative to the residual sum of squares, as determined by the F test

(9) $$F=\left(\mathrm{MSS}/2\right)/\left(\mathrm{RSS}/\left(N‒3\right)\right)$$

where 2 and N-3 are the numbers of degrees of freedom attributed to the model (k = 3 parameters – 1) and to the error term (N – k). The null hypothesis (H_0_) that there is no rhythm (the amplitude is zero) is rejected when F > F_1-α_(2, N-3), where α relates to the chosen probability level for testing H_0_.

**Figure 2:**
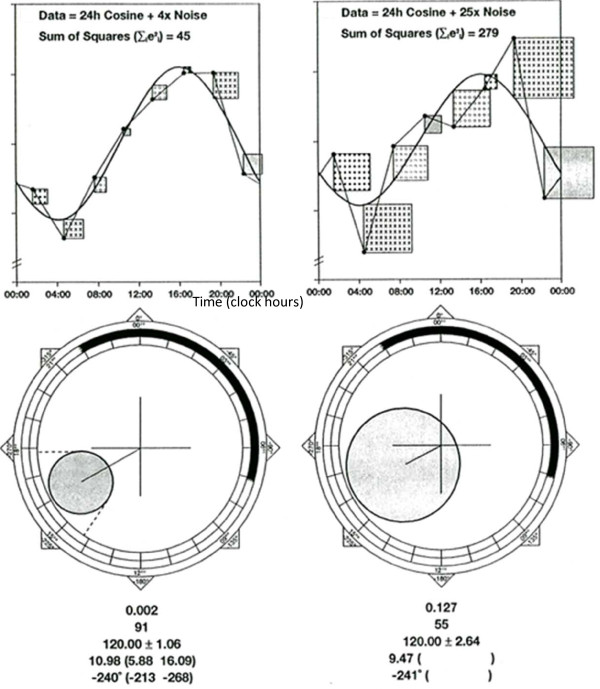
**Single-component single cosinor: hypothesis testing and parameter estimation.** A cosine curve with a given period is fitted to the data (top) by least squares. This approach consists of minimizing the sum of squared deviations between the data and the fitted cosine curve. The larger this residual sum of squares is, the greater the uncertainty of the estimated parameters is. This is illustrated by the elliptical 95% confidence region for the amplitude-acrophase pair (bottom). When the error ellipse does not cover the pole, the zero-amplitude (no-rhythm) test is rejected and the alternative hypothesis holds that a rhythm with the given period is present in the data (left). Conservative 95% confidence limits for the amplitude and acrophase can then be obtained by drawing concentric circles and radii tangent to the error ellipse, respectively. When the error ellipse covers the pole, the null hypothesis of no-rhythm (zero amplitude) is accepted (right). Results (P-value from the zero-amplitude test, percentage rhythm or proportion of the overall variance accounted for by the fitted model, MESOR ± SE, amplitude and 95% confidence limits, acrophase and 95% confidence limits) are listed in each case. © Halberg Chronobiology Center.

For parameter estimation, it seems reasonable to consider the MESOR (M) separately and (β, γ) together. The 1-α confidence interval for $\hat{M}$ is then given by

(10) $$\hat{M}\pm t_{1‒\alpha /2}\left(N‒3\right)\;\hat{\sigma }\;\sqrt{s_{11}^{‒1}}\;$$

where $s_{\mathrm{ij}}^{‒1}$ are the elements of S^-1^,

(11) $$\hat{\sigma }={\left[\mathrm{RSS}/\left(N-3\right)\right]}^{1/2}$$

and t_p_(f) denotes the pth probability point of Student’s t on f degrees of freedom. The covariance matrix for $\left(\hat{\beta },\hat{\gamma }\right)$ is given by

$${\hat{\sigma }}^{2}\left(\begin{array}{l} s_{22}^{‒1}\;s_{23}^{‒1}\\ s_{32}^{‒1}\;s_{33}^{‒1} \end{array}\right),$$

from which a 1-α confidence region for $\left(\hat{\beta },\hat{\gamma }\right)$, or equivalently for $\left(\hat{A},\hat{\phi }\right)$ can be derived. It is based on the F-statistic used for rhythm detection, being evaluated at the estimated value of $\hat{A}$ instead of at A = 0. The resulting equation is that of an ellipse (Figure 2):

(12) $$\sum (x_{i}‒\bar{x}){}^{2}{\left(\beta -\hat{\beta }\right)}^{2}+2\sum \left(x_{i}‒\bar{x}\right)\left(z_{i}‒\bar{z}\right)\left(\beta ‒\hat{\beta }\right)\left(\gamma ‒\hat{\gamma }\right)+\sum {\left(z_{i}‒\bar{z}\right)}^{2}(\gamma ‒\hat{\gamma }){}^{2}\leq 2{\hat{\sigma }}^{2}F_{1‒\alpha }\left(2,N‒3\right)$$

where

$$\bar{X}=\left(\sum_{i}X_{i}\right)/N$$

and

$$\bar{Z}=\left(\sum_{i}Z_{i}\right)/N$$

The region delineated by this ellipse represents the confidence region for the rhythm parameters. Conservative confidence intervals for $\hat{A}$ and $\hat{\phi }$ are obtained by computing the minimal and maximal distances from the pole (zero) to the error ellipse and by drawing tangents from the pole to the error ellipse, respectively [31]. These confidence limits are conservative in the sense that the area they delineate is larger than the confidence ellipse. Limits closer to those corresponding to the α-level chosen for testing can also be computed [43].

Standard errors (SEs) for $\hat{A}$ and $\hat{\phi }$ can also be derived from the covariance matrix for $\left(\hat{\beta },\hat{\gamma }\right)$, by using Taylor’s series expansion:

(13) $$\begin{array}{l} \mathrm{SE}\left(\hat{A}\right)=\hat{\sigma }[s_{22}^{‒1}\cos^{2}\hat{\phi }‒2s_{23}^{‒1}\sin\hat{\phi }\cos\hat{\phi }+s_{33}^{‒1}\sin^{2}\hat{\phi }]{}^{1/2}\\ \mathrm{SE}\left(\hat{\phi }\right)=\hat{\sigma }[s_{22}^{‒1}\sin^{2}\hat{\phi }+2s_{23}^{‒1}\sin\hat{\phi }\cos\hat{\phi }+s_{33}^{‒1}\cos^{2}\hat{\phi }]{}^{1/2}/\hat{A} \end{array}$$

### Regression diagnostic tests

It should be noted that the P-value obtained from the F-test and the corresponding confidence limits derived for $\hat{M},\hat{\beta }\;\mathrm{and}\;\hat{\gamma }$ are valid only if assumptions underlying the use of the least squares procedure are satisfied. These assumptions are (1) the model fits the data well, (2) the residuals are normally distributed, (3) the variance is homogeneous, (4) the residuals are independent, and (5) the parameters do not change over time.

Model adequacy:

Goodness of fit can be examined when replicates are available, either from multiple data collection at different timepoints or from data covering multiple cycles. RSS can then be further partitioned into the “pure error” and the “lack of fit”. An F-test comparing the pure error and lack of fit sums of squares provides a test of the model adequacy [44]. The pure error sum of squares (SSPE) is defined as the sum of squared differences (across all timepoints) between the data collected at a given timepoint and their respective arithmetic mean, whereas the sum of squares ascribed to lack of fit (SSLOF) is obtained by subtracting SSPE from RSS

(14) $$\begin{array}{c} \mathrm{SSLOF}=\mathrm{RSS}‒\mathrm{SSPE}\\ \mathrm{SSPE}=\sum_{i}\sum_{l}{\left(Y_{\mathrm{il}}–{\bar{Y}}_{i}\right)}^{2} \end{array}$$

with ${\bar{Y}}_{i}=\left(\sum_{l}Y_{\mathrm{il}}\right)/n_{i}$ where n_i_ is the number of data collected at time t_i_.

The appropriateness of the model is rejected if

(15) $$F=\left[\mathrm{SSLOF}/\left(m‒1‒2p\right)\right]/\left[\mathrm{SSPE}/\left(N‒m\right)\right]>{F_{1‒}}_{\alpha }\left(m‒1‒2p,N‒m\right)$$

where m is the number of timepoints and p is the number of (cosine) components in the model (p = 1 for the single-component cosinor).

**Figure 3:**
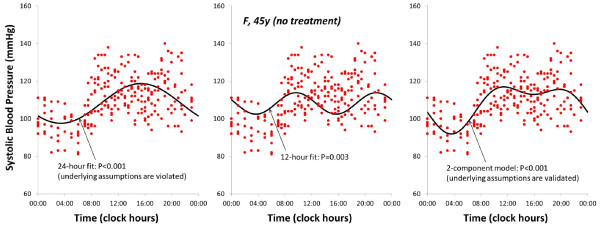
**Multiple-component single cosinor.** Systolic blood pressure data collected over 7 days by a 45-year old woman fitted with a 24-hour cosine curve indicate the presence of lack of fit, departure from normality of residuals, and inhomogeneity of variance (left). The addition of a 12-hour component (middle) to the model (right) yields a better fit for which underlying assumptions are validated. © Halberg Chronobiology Center.

In the presence of lack of fit, adding components in the model may be considered (Figure 3).

Normality of residuals:

The rankit plot provides an attractive visual technique to test normality [44,45]. In this test, the errors (e_i_) are sorted by increasing order and expected values of a normal sample of size N with zero mean (rankits, z_i_) are calculated. If the residuals are normally distributed, the regression of e_i_ on z_i_ is a straight line. The Shapiro-Wilk test of normality can be applied for small sample sizes (N ≤ 50) [46]. For larger sample sizes, a chi-square test of goodness of fit can be used [23] by comparing expected and observed frequencies of residuals grouped in classes.

Homogeneity of variance:

The variance of the error term sometimes depends on the expected level of the variable examined. This can be the case of hormonal data such as melatonin that assumes large values by night but only very low values during the day. All day-time values being very small, the variance is also small. But as night-time data can vary greatly, their variance is also inflated. Deviation from variance homogeneity can be revealed by a plot of residuals as a function of the fitted values [47]. A horizontal band around zero in such a plot indicates that the assumption is valid. If it is violated, data can be transformed, for instance by taking their square root or their logarithm. A numerical test can also be performed by fitting the model to the square of the estimated values instead of the data in order to obtain residuals re_i_. If

(16) $$F=\left(N‒2p‒2\right)r^{2}/\left(1‒r^{2}\right)>{F_{1‒}}_{\alpha }\left(1,N‒2p‒2\right)$$

where r denotes the regression coefficient of re_i_ on e_i_, the assumption of homogeneity of variance is rejected.

Independence of residuals:

While violation of independence usually does not affect the estimate of the parameter themselves, their confidence intervals tend to be under-estimated [48]. When residuals are positively correlated, they tend to assume the same sign for long sequences. The runs test is a non-parametric test that allows to test whether sequences (runs) of positive and negative residuals occur randomly. Specifically, if successive errors are independent, there cannot be any regular sequences, either too long or too short. In other words, the number of runs cannot be too small or too large, respectively. For a given sample size, tables list limits for acceptable numbers of runs compatible with the assumption of independence [23,49,50].

When residuals are correlated, the data can either be low-passed filtered by averaging or decimation (using only one every k values, thereby lengthening the sampling interval from Δt to kΔt). A slightly different model can also be considered wherein the error term is replaced by an autoregressive error term [10,51,52].

Stationarity:

The problem of stationarity arises primarily in long time series, when the MESOR, amplitude, acrophase and/or period can change as a function of time. This may occur, for instance, when a person being monitored travels across time zones (e.g., from the USA to Europe). A head cold or pain can also bring about transient changes in circadian rhythm characteristics of variables such as body temperature, blood pressure or heart rate. Changes in period can be anticipated when time clues (environmental synchronizers) are removed. They are also present in the case of components with no strong environmental synchronizer. This concerns primarily non-photic cycles such as the about 1.3-year transyear and the about 5-month cis-half-year found in solar wind speed and solar flares, respectively. These components are wobbly by nature, even in the environment. Counterparts in biology have been found, as discussed elsewhere [3,53-56]. There are several approaches available to analyze non-stationary time series, such as wavelets, short-term Fourier transforms, and gliding spectral windows complemented by chronobiologic serial sections, as discussed below.

For short and sparse time series, for which the cosinor method was originally developed, underlying assumptions are usually valid (or at least statistical power is not sufficient to invalidate them). With longer and denser data, the likelihood of violating one or several underlying assumptions increases. Violation of one assumption can also result in violating one or several other assumptions. For instance, when there is lack of fit, residuals tend not to be independent and may not follow a normal distribution. Confidence intervals and P-values tend to be affected more than the estimation of parameters. Even when one or several underlying assumptions are violated, the information from the cosinor analysis may be of value as long as results are properly qualified. Many of the conventional methods of data analysis depend on similar assumptions, with the exception of robust non-parametric techniques [10,57-61].

### Multiple-component cosinor

The single-component cosinor is easily extended to a multiple-component model (Figure 3)

(17) $$Y\left(t\right)=M+\sum_{{}_{j}}A_{j}\cos\left(2\mathrm{\pi t}/\tau_{j}+\phi_{j}\right)+e\left(t\right),j=1,2,\ldots ,p$$

Instead of solving a system of 3 equations in 3 unknowns, there are 2p + 1 normal equations to estimate M and p pairs of (β_j_, γ_j_) or (A_j_, ϕ_j_) when τ_j_ are assumed known. Generally, in the normal equations d = Su

(18) $$\begin{array}{l} d=\sum_{{}_{i}}{x_{i}}_{v}Y_{i}\mathrm{and}\;\mathrm{Su}=\sum_{j}u_{j}\sum_{i}{x_{i}}_{v}x_{\mathrm{ij}}\mathrm{for}\;i=1,2,\ldots ,N;\\ j=1,2,\ldots ,2p+1;\mathrm{and}\;v=1,2,\ldots ,2p+1 \end{array}$$

where {x_ij_} are the cos(2πt_i_/τ_j_) and sin(2πt_i_/τ_j_).

Estimates of u (M, β_1_, γ_1_, β_2_, γ_2_, …, β_p_, γ_p_) are obtained as

$$\;\hat{u}=S^{‒1}X’Y\;\mathrm{where}\;S=X’X=|\sum_{i}x_{\mathrm{ij}}x_{\mathrm{ik}}|.$$

A confidence ellipsoid can be determined [43] from which approximate confidence intervals can be derived for each component’s amplitude and acrophase, as outlined above. Computations are greatly simplified in the case of equidistant data covering an integer number of cycles [45].

A multiple-component model is useful to obtain a better approximation of the signal’s waveform when it deviates from sinusoidality. For instance, a 2-component model consisting of cosine curves with periods of 24 and 12 hours has been extensively used to analyze ambulatory blood pressure data (Figure 3) [62-64]. On the average, these two components account for the larger overall variance in this case [65]. This model is usually well-suited to approximate the nightly drop in blood pressure that reaches a nadir around mid-sleep [66], the slight increase thereafter and a sharper increase after awakening, the post-prandial dip seen more prominently in the elderly [67], and a slow decline in the evening. Whereas better-fitting models can be obtained for each individual patient, the choice of a given model used as a reference standard makes it possible to derive reference values (such as 90% prediction limits) for the model’s parameters for specified populations, usually clinically healthy men or women in several age groups [62]. Deviations from these norms can then be viewed as indicative of rhythm alteration. In addition to the well-known cardiovascular disease risk associated with an elevated blood pressure MESOR, outcome studies [68-72] have determined that certain other rhythm alterations affecting the circadian amplitude and acrophase are also associated with an increase in cardiovascular disease risk [64].

### Population-mean cosinor

When data are collected as a function of time on 3 or more individuals, the population-mean cosinor procedure renders it possible to make inferences concerning a population rhythm, provided the k individuals represent a random sample from that population. Each individual series is analyzed by the single- or multiple-component single cosinor to yield estimates of $\left\{\hat{u}={\hat{M}}_{i},{\hat{\beta }}_{1i},{\hat{\gamma }}_{1i},{\hat{\beta }}_{2i},{\hat{\gamma }}_{2i},\ldots ,{\hat{\beta }}_{\mathrm{pi}},{\hat{\gamma }}_{\mathrm{pi}},i=1,2,\ldots ,k\right\}$. The goal is to make inferences concerning the population averages of the parameters, u*. The “*” indicates that the expectations are population averages and not averages over the k individuals sampled. Individual vectors u_i_ are assumed to represent a random sample from a (2p + 1)-variate, normal population with mean u*. The within-individual variances are also assumed to be equal, so that the pooled estimate of variance can be estimated as

(19) $${\hat{\sigma }}^{2}=\sum_{j}\left(n_{j}‒\left(2p+1\right)\right){\hat{\sigma }}_{j}^{2}/\left(N‒\left(2p+1\right)k\right)$$

When the sample sizes for all individuals are the same or almost the same, as is often the case in hybrid designs, the population estimates are unweighted averages of the individual parameters

(20) $$\begin{array}{l} \hat{u}*=\sum_{j}{\hat{u}}_{\mathrm{jn}}/k\;\mathrm{for}\;j=1,2,\ldots ,k\;\mathrm{and}\\ n=1,2,\ldots ,2p+1\left({\hat{u}}_{n}=\left\{\hat{M},{\hat{\beta }}_{1},{\hat{\gamma }}_{1},{\hat{\beta }}_{2},{\hat{\gamma }}_{2},\ldots ,{\hat{\beta }}_{p},{\hat{\gamma }}_{p}\right\}\right) \end{array}$$

and the population amplitudes and acrophases can be estimated using the relations

$$\hat{\beta }*=\hat{A}*\cos\hat{\phi }*;\hat{\gamma }*=‒\hat{A}*\sin\hat{\phi }*$$

In the above conditions and assuming normality of errors and individual parameters, sample variances can be computed as

(21) $$\begin{array}{l} {\hat{\sigma }}_{M}^{2}=\Sigma_{j}{\left({\hat{M}}_{j}-\hat{M}*\right)}^{2}/\left(k‒1\right);{\hat{\sigma }}_{\beta n}^{2}=\Sigma_{j}{\left({\hat{\beta }}_{\mathrm{nj}}-\hat{\beta }*\right)}^{2}/(k‒1);\\ {\hat{\sigma }}_{\gamma n}^{2}=\Sigma_{j}{\left({\hat{\gamma }}_{\mathrm{nj}}‒\hat{\gamma }*\right)}^{2}/(k‒1);{\hat{\sigma }}_{M\beta 1}^{2}=\Sigma_{j}\left({\hat{M}}_{j}‒\hat{M}*\right)({\hat{\beta }}_{1j}‒{\hat{\beta }}_{1}*)/(k‒1);\\ \;\text{and}\;\text{similarly}\;\text{for}\;\text{the}\;\text{other}\;\text{cross}‒\text{products} \end{array}$$

where

j=1,2,…,kandn=1,2,…,p

In the case when the population-mean cosinor can be applied separately for each trial period (p = 1), a confidence interval for M* is given by

(22) $$\hat{M}*\pm {t_{1‒}}_{\alpha /2}\left(k‒1\right){\hat{\sigma }}_{M}/k^{1/2}$$

and a joint 1-α confidence ellipse for $\left(\hat{\beta }*,\hat{\gamma }*\right)$ consists of all points (β_z_*,γ_z_*) satisfying

(23) $$\begin{array}{l} {\left(\beta_{z}*‒\hat{\beta }*\right)}^{2}/{\hat{\sigma }}_{\beta }^{2}‒2r\left(\beta_{z}‒\hat{\beta }*\right)\left(\gamma_{z}*‒\hat{\gamma }*\right)/{\hat{\sigma }}_{\beta }{\hat{\sigma }}_{\gamma }+{\left(\gamma_{z}*‒\hat{\gamma }*\right)}^{2}/{\hat{\sigma }}_{\gamma }^{2}\leq \\ 2\left(1‒r^{2}\right)\left(k‒1\right){F_{1‒}}_{\alpha }\left(2,k‒2\right)/\left(k\left(k‒2\right)\right) \end{array}$$

where

$$r={\hat{\sigma }}_{\beta \gamma }/\left({\hat{\sigma }}_{\beta }{\hat{\sigma }}_{\gamma }\right)$$

**Figure 4:**
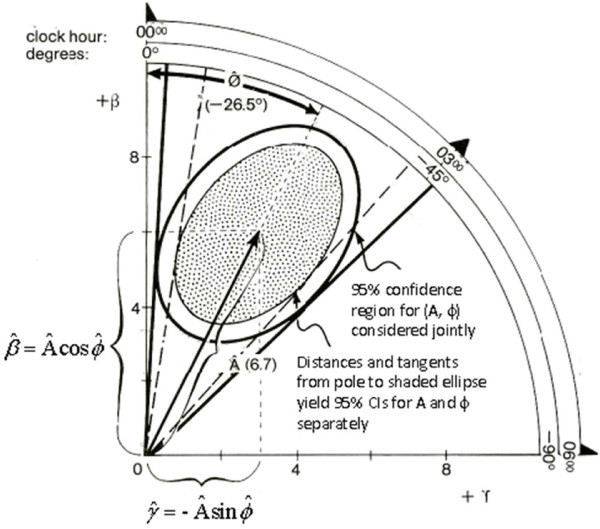
**Elliptical confidence limits.** The outer ellipse delineates the 95% confidence region for the joint estimation of the amplitude and acrophase (as a pair). Distances and tangents drawn from the pole to this outer ellipse yield conservative 95% confidence limits for the amplitude and acrophase considered separately as the area thus delineated is larger than the area of the outer ellipse. In order to obtain separate 95% confidence limits for the amplitude and acrophase, distances and tangents need to be drawn to a somewhat smaller (inner) ellipse. For further details, see [43]. © Halberg Chronobiology Center.

The null hypothesis of A* = 0 is rejected if

(24) $$\left[\left(k\left(k‒2\right)\right)/\left(2\left(k‒1\right)\right)\right]\left[1/\left(1‒r^{2}\right)\right]\left[\hat{\beta }{*}^{2}/{\hat{\sigma }}_{\beta }^{2}‒2r\hat{\beta }*\hat{\gamma }*/{\hat{\sigma }}_{\hat{\beta }}{\hat{\sigma }}_{\gamma }+\hat{\gamma }{*}^{2}/{\hat{\sigma }}_{\gamma }^{2}\right]>{F_{1‒}}_{\alpha }\left(2,k‒2\right)$$

and approximate confidence intervals for $\hat{A}*$ and $\hat{\phi }*$ can be obtained by computing the minimal and maximal distances from the pole (zero) to the error ellipse and by drawing tangents from the pole to the error ellipse, respectively (Figure 4). As for the single cosinor, closer approximate limits can also be computed [43].

### Parameter tests

Test statistics have been developed to test the equality of MESORs, amplitudes and acrophases considered jointly or separately for the case of the single cosinor and the population-mean cosinor [43]. These tests can allow for a clearer interpretation of the results, for instance in a circadian experiment involving 6 timepoints 4 hours apart: Student t-tests are sometimes applied at each separate timepoint without adjustment of the P-values for multiple testing; when differences in opposite directions are found, parameter tests may reveal a difference in the circadian amplitude in the absence of a difference in MESOR or in the circadian acrophase [73].

### Least squares spectra and population-mean cosinor spectra

The circadian rhythm is often prominent. It is also ubiquitous. These features enabled the single cosinor procedure to be applied to many short data series covering no more than a single cycle in order to yield valuable information regarding the organization of the circadian system in different species. Computationally, estimates of the MESOR, amplitude and acrophase can be obtained for any trial period. This procedure, however, is valid only if there is sufficient evidence for considering this particular trial period. In the absence of such evidence, results can no longer be taken at their face value.

It has become much easier for chronobiologists to collect data over much longer spans and/or at much shorter intervals, but it has been more difficult to obtain series of equidistant data. Even for variables that are obtained with automated instrumentation (such as telemetry or ambulatory blood pressure monitors), it is not uncommon to have missing data or to have additional data collected manually at times different from the scheduled times. Investigations have also extended outside the circadian realm. For these reasons, a least squares approach to time series analysis remains attractive, as long as caution is properly taken in interpreting the results.

Just as a chronogram provides useful information prior to quantitative data analysis, a view of the time structure of the data in the frequency domain can also be informative. For this purpose, using the cosinor at Fourier frequencies in the range of 1/T (where T is the length of the data series) up to 1/2Δt (where Δt is the sampling interval) can be viewed as no more than another macroscopic view of the data. A plot of amplitudes as a function of frequency (least squares spectrum) is equivalent to a discrete Fourier transform when data are equidistant [38].

**Figure 5:**
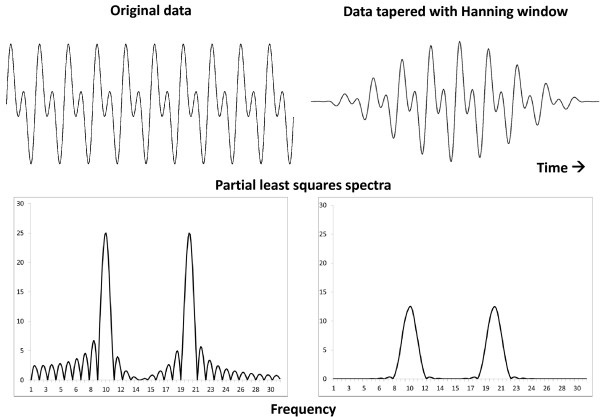
**Effect of applying a Hanning taper on the least squares spectrum.** A simulated signal consisting of a fundamental and second harmonic of equal amplitudes sampled over 10 cycles (top left) is tapered with a Hanning window (top right). Corresponding least squares spectra (bottom) indicate that while the spectral location of the two peaks remains the same, the amplitudes are reduced and the bandwidths are wider. Sidelobes are also greatly diminished. Simulation and original drawings from C. Lee-Gierke. © Halberg Chronobiology Center.

– Large spectral peaks indicate the presence of signals and provide an approximate estimate of their periods. This information can be used to validate anticipated components while also revealing the presence of other cycles. For rhythms that are anticipated, rhythm detection and parameter estimation can proceed as outlined above as long as P-values are adjusted for multiple testing [74]. Caution needs to be taken regarding non-anticipated cycles. The information thus gained can be used to design the next study or to examine other similar data series that could serve as replications. Additional analyses can be performed to determine the extent of stability of the unanticipated component, for instance by means of applying a chronobiologic serial section [21] or a gliding spectral window [75].

– Plotting log-amplitudes versus log-frequency provides useful information regarding the noise structure [65]. White noise corresponds to about the same background amplitudes across the entire frequency range. Larger background amplitudes at lower than at higher frequencies represents colored (or correlated) noise, indicating that underlying assumptions are not met, resulting in under-estimated P-values and too-liberal confidence intervals of rhythm parameters. The noise structure can in itself be valuable. It is used for instance in assessing the 1/f behavior of heart rate variability [76].

– Single spectral peaks are found only if the data cover an integer number of cycles. If this is not the case, the signal spreads over several spectral lines [10]. When this happens and the underlying signal was anticipated, it is possible to determine the period (frequency) corresponding to the maximal amplitude by applying the single cosinor procedure not only at the Fourier frequencies but at additional intermediary frequencies as well. Whereas this may provide a clearer picture of the signal, it should be realized that the resolution in frequency (1/T) remains the same, being determined by the series length, T. Tapers such as a Hanning window [77] can be used to reduce sidelobes associated with the finite observation span, but this procedure also affects the estimation of the rhythm parameters. While a Hanning taper does not affect the location of spectral peaks in a spectrum, the width of the peak is wider and the amplitude is reduced (Figure 5). It remains useful, however, for a macroscopic view of the time structure of the data.

Least squares spectra can be very helpful in exploratory analyses, but it should be realized that assumptions underlying the use of the single cosinor (notably independence and normality) are violated more often than not. Population-mean cosinor spectra are a useful complementary approach not prone to this limitation. This method is similar to the power spectrum obtained by smoothing the periodogram, which is more reliable for testing unknown periodicities [78], with the important difference, however, of retaining the phase information. The averaging (smoothing) can be done either in the frequency domain by averaging across consecutive Fourier frequencies, or in the time domain. The population-mean cosinor spectrum uses averaging in the time domain. The method consists of subdividing the observation span T into several (e.g., k) subsections (or intervals, I) of equal length (I = T/k). A least squares spectrum is computed for each interval, using the same common reference time. The population-mean cosinor procedure is then applied at each trial frequency to summarize results across the k intervals. The procedure can be repeated by using different values of k. Unknown signals consistently detected by this approach may thus be viewed with added confidence.

### Extended linear-nonlinear cosinor

When the period is unknown, the single cosinor model (Equations 1 and 12) can no longer be linearized in its parameters as the period is in the argument of the cosine function. Starting from an initial (guess) estimate for the period, all parameters can be estimated using iterations aimed at minimizing the residual sum of squares. Marquardt [79] developed an algorithm which performs an optimum interpolation between the Taylor series and gradient methods. He also derived a way to approximate confidence intervals for all parameters, including the period [80]. For the particular case of single-component models, Bingham offers an easily understood approach [81].

For low-frequency signals, simulated annealing [82] is another suitable method that has the advantage of not requiring the specification of initial values for the periods. This approach does not perform well, however, for very sharp signals in the higher frequency range of the spectrum. Both simulated annealing and Marquardt’s nonlinear approach performed best in distinguishing two signals with close periods sampled over less than a beat cycle, when compared to other approaches [83].

For signals with a symmetrical waveform, the nonlinear procedure can yield an acceptable estimate of the fundamental period on the basis of very short records not even covering a full cycle [84]. This is not the case, however, when the waveform is asymmetrical. Simulations indicate that about 5 cycles are needed to obtain a reasonable estimate of the period in this case, when the model fitted includes only the fundamental component. Including additional harmonic terms in the model allows the nonlinear procedure to correctly estimate the fundamental period with data covering no more than 2 cycles [84].

### Analysis of non-stationary data

**Figure 6:**
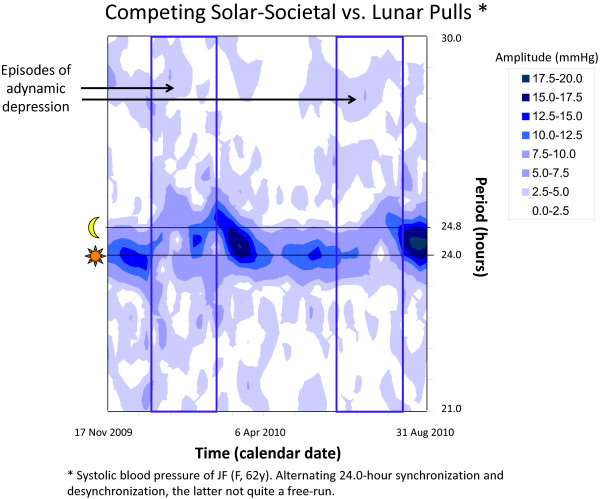
**Gliding spectral window (surface chart).** Systolic blood pressure was automatically measured around the clock by a 62-year old woman with recurring episodes of adynamic depression occurring twice a year and lasting 2–3 months. Complementary nonlinear analyses (not shown) indicate the coexistence of about 24.0- and 24.8-hour components, their relative prominence alternating between wellness and adynamic depression. Changes in the most prominent circadian period as a function of time are apparent from the changes in amplitude (shading) and location along the vertical scale. © Halberg Chronobiology Center.

When data are equidistant or rendered equidistant by averaging and filling data gaps by interpolation, wavelets can be performed [85]. This approach has been useful to uncover components not detected earlier [86]. Short-term Fourier transforms can be used to visualize changes in the spectral structure of the data as a function of time [87]. Alternatively, gliding spectral windows [75] can be computed. The method consists of defining an interval (I) that is progressively displaced by a given increment (δt) throughout the data series. A least squares spectrum is computed over each interval over a specified frequency range. Both a fractional harmonic increment (δh < 1) and overlapping intervals (δt < I) are chosen to help visualize the time course of changes in frequency and/or amplitude occurring as a function of time in a 3D graph and/or a surface chart. In such a display, time and frequency are the two horizontal axes and amplitude is shown on the vertical axis in a 3D plot or as different shadings in a surface chart. One example relates to competing about 24.0- and 24.8-hour components coexisting in the physiology of an apparently seleno-sensitive woman with adynamic episodes recurring twice a year and lasting 2–3 months, as illustrated for systolic blood pressure in Figure 6. Another example illustrates the changing prominence of the about-weekly and about-daily rhythms in blood pressure and heart rate during the first 40 days of life of a clinically healthy boy [88]. Whereas the procedure can be performed on non-equidistant data, the interpretation of results is greatly helped when data are equidistant, as changes in sampling rate are also associated with changes in spectral structure appearing on the graph. A judicious choice of I and of the frequency range examined is important in order to minimize sidelobes. The use of a Hanning taper [77] is also helpful in this kind of exploratory analysis.

**Figure 7:**
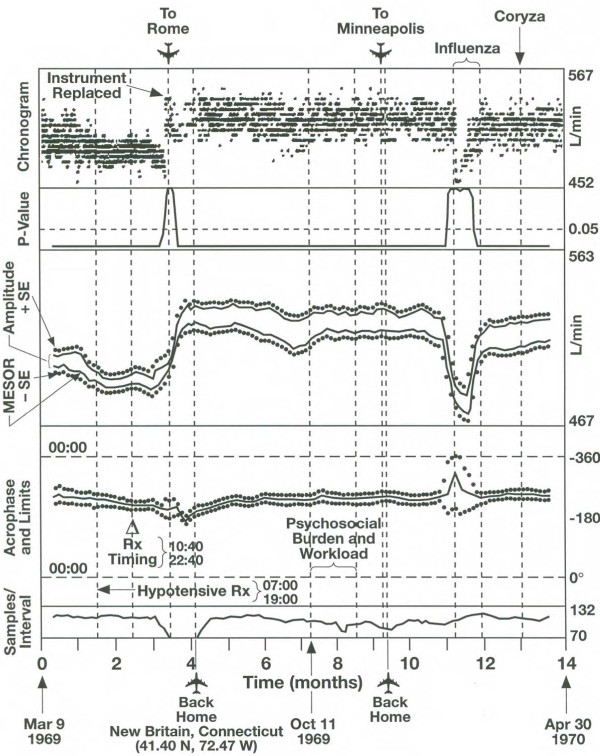
**Chronobiologic serial section.** Peak expiratory flow was self-measured several times a day by a 53-year old man. The data covering a 14-month span are shown in row 1. They are analyzed in a 20-day interval progressively displaced by 2 days. Data in each interval are fitted with a 24-hour cosine curve. From the P-values shown in row 2, it can be seen that the circadian rhythm was detected with statistical significance most of the time, except for two short spans, one coinciding with a transmeridian flight (when fewer data were collected, row 5) and the other with influenza. Whereas the 24-hour acrophase remains relatively stable throughout the record (row 4), the MESOR (row 3, lower curve) and to a lesser extent the circadian amplitude (row 3, distance between the two curves) undergo sharp changes, notably in association with the influenza and earlier with a change in treatment timing. © Halberg Chronobiology Center.

Whenever focus can be placed on a specified component with a given trial period, a chronobiologic serial section [21] can be performed. As for the gliding spectral window, an interval I is selected that is progressively displaced throughout the time series by δt increments. To data in each interval, the single-component single cosinor procedure is applied. To visualize the results, a chronogram is shown on top, followed by the sequence of MESORs, amplitudes and acrophases as they change as a function of time, provided with a measure of uncertainty. Corresponding P-values from the zero-amplitude test and the number of data per interval are also displayed to help interpret any change in the results. This procedure has been extensively used in studies of phase shifts associated with transmeridian flights [89], as illustrated in Figure 7, and in cases when the circadian rhythm is desynchronized from 24 hours [90,91].

**Figure 8:**
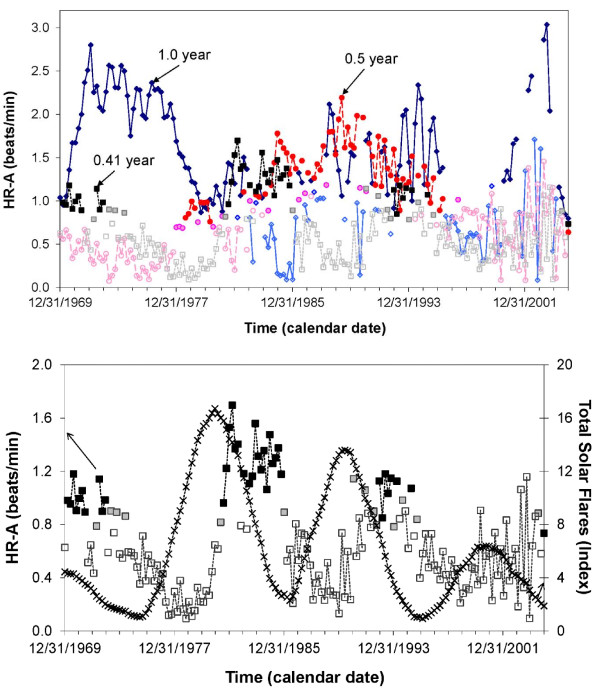
**Multiple-component serial section.** Heart rate, self-measured a few times each day by a healthy man over several decades, was averaged over consecutive weeks. The weekly averages are fitted with a 3-component model consisting of cosine curves with periods of 1.0, 0.5, and 0.41 year in a 4-year interval moved by 0.2 year throughout the entire record. The time course of amplitudes of the 3 components is shown on top. Solid-filled, light-filled and empty symbols correspond to P-values <0.05, 0.05 < P < 0.10, and >0.10 from the zero-amplitude tests, respectively. Results for the 0.41-year component are reproduced below, where they are compared with the solar flare index that had been reported by physicists to be characterized by an about 5-month (0.41-year) component. The prominence of the about 5-month component in human heart rate follows an about 11-year cycle, which is similar to that characterizing solar flares. © Halberg Chronobiology Center.

**Figure 9:**
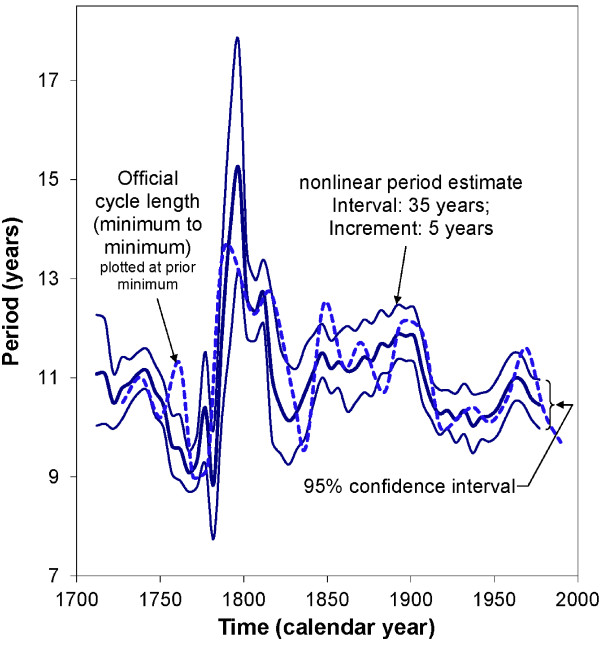
**Nonlinear serial section.** The period of the about 11-year cycle in solar activity is estimated by nonlinear least squares applied to yearly Wolf numbers analyzed in a 35-year interval progressively moved by 5 years throughout the time series. The solar activity cycle has a period that can vary from about 9 to 15 years, shown here with its 95% confidence interval and compared with the official cycle length. © Halberg Chronobiology Center.

The procedure has been extended in two ways. First, a multiple-component instead of a single-component single cosinor model can be fitted in each interval. This procedure has been used for instance to illustrate that the prominence of an about 5-month component of heart rate self-measured over 4 decades by a clinically healthy man follows the about 11-year change in solar flares in which this component had been documented [92]. In this analysis, the 5-month component was fitted together with 1.0- and 0.5-year components over a 4-year interval displaced by 0.2-year increments [93]. Figure 8 illustrates the changing prominence of the three components as a function of time. An about 11-year cycle in the prominence of the about 5-month component is highlighted. Second, a nonlinear model can be fitted in each interval and the period displayed as a function of time with its 95% confidence interval. This approach was used to illustrate the great variability in the length of the about 11-year solar activity cycle, Figure 9[94].

## Discussion and conclusion

There are, of course, other important tools for the analysis of time series [95-103]. Most of them, however, require that the data be equidistant. This overview focused specifically on the use of the cosinor and its different extensions. The method is fairly simple and its results lead to meaningful interpretations. Despite its several shortcomings related primarily to the difficulty of satisfying all assumptions underlying the use of regression techniques, its wide-ranging applications have played an important role in the development of chronobiology as a quantitative scientific discipline. Used with caution, results based on a combination of exploratory analyses with the different cosinor routines and other conventional statistical tests, progress has also been made in the field of chronomics which aims at mapping broad time structures from the high-frequency brain waves to the multi-decadal cycles characterizing space-terrestrial weather influencing human physiology and pathology [3,104].

Despite its simplicity, some reluctance remains for some investigators to use the cosinor for estimating rhythm parameters or for considering more than a single test time in designing experiments. Too many studies still rely on testing only at a fixed time of day (to control for the circadian variation) or at most at two times 12 hours apart, ignoring the possibility that the two selected timepoints may be at the midline crossings rather than at the peak and trough where differences are maximal. As discussed elsewhere, such practice can be misleading in missing an existing difference or even in finding a difference in mean when none exists [15-17]. Computing day-night differences in lieu of an amplitude and acrophase is also widely done to interpret ambulatory blood pressure monitoring records in terms of “dipping” [105], despite the documentation in several outcome studies of the superiority of a chronobiological approach [106,107].

To some extent, this status quo may be accounted for by the lack of dissemination of computer software offering chronobiologists tools for time series analysis applicable to non-equidistant data. This situation is slowly changing, however. Personal computers have become more powerful and statistical packages have become more readily available for relatively easy use by investigators not necessarily versed in all statistical details underlying the programs included in the software packages. While professional statistical software packages remain somewhat expensive for individual users, several open-source packages (such as Octave and R) offer an attractive alternative, notably since some are platform-independent, running on PCs, Macs or Linux systems [108]. Programmers have taken advantage of the tools available in these packages to write code to perform analytical tasks of interest to chronobiologists. Perhaps the most comprehensive package is that developed by Oehlert and Bingham [109], offering a large array of procedures that can be applied by writing minimal coding instructions to call the different macros. Selected programs used in chronobiology have long been offered on the website of Refinetti [110], with clear instructions on how to run the programs. While not open-source, the Expert Soft Technologie website [111] also offers an array of cosinor-based and other procedures, including techniques for the study of non-stationary signals. These programs have been used in the study of shift-workers [112].

In summary, selected methods for the study of biologic time series have been reviewed and their relative merits have been discussed in the light of underlying assumptions. Some illustrative applications have also been mentioned. When the choice of a model is justified, and it is functional and explicative, quantitative methods of data analysis are extremely valuable to specify the model and obtain estimates of its parameters. Even when underlying assumptions are not fully met, point estimates of the parameters can be very useful. More caution is needed, however, in deciding whether P-values and confidence intervals are trustworthy, since violation of underlying assumptions tends to yield results that are too liberal. Once this limitation is taken into consideration, data analysis methods as described herein constitute extremely valuable tools for research in chronobiology and chronomics.

## Competing interests

The author declares that she has no competing interests.

## Support

Halberg Chronobiology Fund, University of Minnesota Supercomputing Institute.
